# Profile of copper-associated DNA methylation and its association with incident acute coronary syndrome

**DOI:** 10.1186/s13148-021-01004-w

**Published:** 2021-01-27

**Authors:** Pinpin Long, Qiuhong Wang, Yizhi Zhang, Xiaoyan Zhu, Kuai Yu, Haijing Jiang, Xuezhen Liu, Min Zhou, Yu Yuan, Kang Liu, Jing Jiang, Xiaomin Zhang, Meian He, Huan Guo, Weihong Chen, Jing Yuan, Longxian Cheng, Liming Liang, Tangchun Wu

**Affiliations:** 1 Department of Occupational and Environmental Health, Key Laboratory of Environment and Health, Ministry of Education and State Key Laboratory of Environmental Health (Incubating), School of Public Health, Tongji Medical College, Huazhong University of Science and Technology, 13 Hangkong Rd., Wuhan, 430030 Hubei China; 2 Suzhou Center for Disease Prevention and Control, Suzhou, China; 3 Department of Cardiology, Union Hospital, Tongji Medical College, Huazhong University of Science and Technology, Wuhan, China; 4 Department of Epidemiology, Harvard T.H. Chan School of Public Health, Boston, MA USA; 5 Department of Biostatistics, Harvard T.H. Chan School of Public Health, Boston, MA USA

**Keywords:** Copper, DNA methylation, Acute coronary syndrome, Gene expression

## Abstract

**Background:**

Acute coronary syndrome (ACS) is a cardiac emergency with high mortality. Exposure to high copper (Cu) concentration has been linked to ACS. However, whether DNA methylation contributes to the association between Cu and ACS is unclear.

**Methods:**

We measured methylation level at > 485,000 cytosine-phosphoguanine sites (CpGs) of blood leukocytes using Human Methylation 450 Bead Chip and conducted a genome-wide meta-analysis of plasma Cu in a total of 1243 Chinese individuals. For plasma Cu-related CpGs, we evaluated their associations with the expression of nearby genes as well as major cardiovascular risk factors. Furthermore, we examined their longitudinal associations with incident ACS in the nested case-control study.

**Results:**

We identified four novel Cu-associated CpGs (cg20995564, cg18608055, cg26470501 and cg05825244) within a 5% false discovery rate (*FDR*). DNA methylation level of cg18608055, cg26470501, and cg05825244 also showed significant correlations with expressions of *SBNO2*, *BCL3*, and *EBF4* gene, respectively. Higher DNA methylation level at cg05825244 locus was associated with lower high-density lipoprotein cholesterol level and higher C-reactive protein level. Furthermore, we demonstrated that higher cg05825244 methylation level was associated with increased risk of ACS (odds ratio [OR], 1.23; 95% CI 1.02–1.48; *P* = 0.03).

**Conclusions:**

We identified novel DNA methylation alterations associated with plasma Cu in Chinese populations and linked these loci to risk of ACS, providing new insights into the regulation of gene expression by Cu-related DNA methylation and suggesting a role for DNA methylation in the association between copper and ACS.

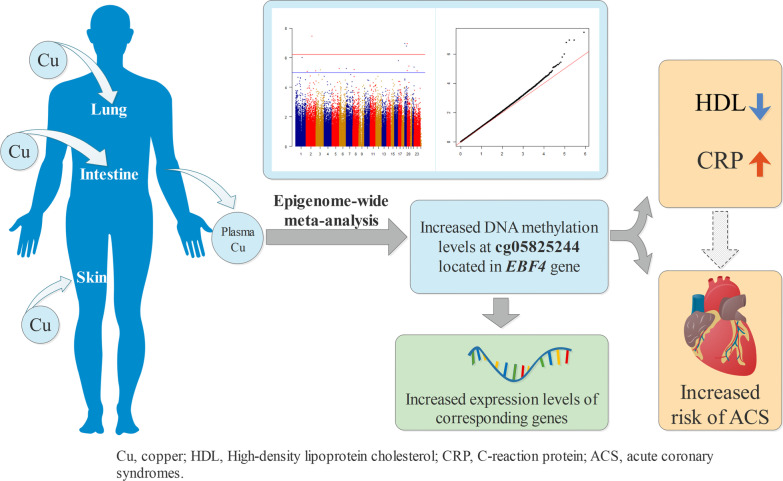

## Introduction

Copper (Cu) is an essential metal for the human body and involves in the regulation of biological metabolism and enzyme synthesis, whereas excessive Cu can also be detrimental to human health [1]. Epidemiological studies have shown that exposure to high copper concentration was associated with an increased risk of cardiovascular disease (CVD) [2–4]. Acute coronary syndrome (ACS) is a severe CVD subtype with high mortality, including acute myocardial infarction (AMI) and unstable angina (UA). A previous meta-analysis of 19 case-control studies showed a significant positive association between high serum Cu level and myocardial infarction [5]. In a prospective cohort study of 1666 participants in eastern Finland, researchers observed that serum copper concentration in the two highest tertiles associated with 3.5-fold and 4.0-fold risk of AMI [6]. Although the underlying mechanisms between high copper status and risk of ACS were not fully understood, accumulating evidence indicated that Cu exposure can cause the production of oxidative stress products (including reactive oxygen species) [7, 8], pro-inflammatory factors [9], abnormal blood lipid metabolism [10], and endothelial cell damage [11].

DNA methylation is a process by which methyl groups are added to the DNA molecule without altering the primary DNA sequence [12]. It is believed that DNA methylation can participate in multiple cellular, physiological, biochemical, and pathological processes by regulating gene expression and genomic stability [13]. DNA methylation has been associated with a range of adverse health outcomes, including ACS [14], cancer [15], diabetes [16], and neurodegenerative diseases [17]. Furthermore, DNA methylation is the most well-studied epigenetic regulatory factor related to metal exposure, and previous studies have suggested that methylation changes related to metal exposure may provide key insights into disease development and susceptibility [18–21]. For example, Everson et al. demonstrated that cadmium-associated differential methylation may be a potentially novel mechanism for adverse effects of prenatal cadmium on the offspring [18]. A recent study has found that mercury-associated alterations in methylation were associated with adverse neurological outcomes [19]. Ren et al. [20] have reviewed that alteration in methylation may mediate the toxicity and carcinogenicity caused by arsenic exposure.

DNA methylation may also be one of the important mechanisms by which Cu exposure exerts its biological effects on ACS. Investigating DNA methylation alterations related to Cu is important to understand the metabolism and pathogenesis of copper. A study comprising 126 elder individuals indicated that the daily intake of Cu had a negative correlation with the global DNA methylation profile of leukocytes [22]. To date, only one genome-wide DNA methylation study has been conducted to investigate associations between placental Cu and DNA methylation alterations in humans, and they observed nine Cu-associated differentially methylated regions (DMRs) and 15 suggestive cytosine-phosphoguanine sites (CpGs) (*p* Values < 1 × 10^−5^) [23]. So far, no studies have been conducted to identify gene-specific epigenetic alterations related to plasma Cu in middle-aged and elderly populations and investigate their relations to risk of ACS. To fill a gap in this field, we performed an epigenome-wide association study (EWAS) of plasma Cu across five Chinese panels and assessed the association of findings with gene expressions, cardiovascular risk factors and incident ACS.

## Materials and methods

### Study design and participants

We conducted a genome-wide meta-analysis of DNA methylation and plasma Cu concentrations across five panels in Chinese populations, four of which have been described in detail previously [24, 25]. The detailed description of the study population was shown in Additional file 1: Method S1.

Briefly, a nested case-control study was conducted by randomly selected 344 incident ACS cases and 344 controls matched for age (± 3 years), sex, and sampling time at baseline (± 40 days), based on baseline and the first follow-up of the Dongfeng-Tongji (DFTJ) cohort, a prospective cohort recruited retired workers from Dongfeng Motor Corporation (DMC) in Shiyan City, Hubei Province, China [26]. Totally, 688 individuals were included in this study as DFTJ panel. The ascertainment criteria for incident ACS were the first occurrence of unstable angina (UA), non-ST-segment elevation myocardial infarction (NSTEMI), or STEMI during follow-up [27]. ACS was confirmed and classified according to the diagnostic criteria of AHA and WHO [28–30] and finally diagnosed by professional physicians.

A two-stage genome-wide methylation association analysis was performed to identify the DNA methylation alterations associated with prevalent ACS [25]. This study included 103 prevalent ACS patients in Wuhan (ACS-WH panel) and 103 prevalent ACS patients in Zhuhai (ACS-GD panel). A total of 283 controls for the prevalent ACS patients were selected from the Wuhan-Zhuhai (WHZH) cohort, a cohort recruited community residents (18–80 years old) lived in Wuhan or Zhuhai city for over 5 years [31]. These individuals were included in our study as WHZH panel.

A total of 144 healthy individuals who had regular physical examinations at the Health Examination Center of Dongfeng Central Hospital (Dongfeng Motor Corporation and Hubei University of Medicine) in Shiyan were recruited to investigate whether DNA methylation was associated with gene expression. These individuals were included in our study as SY panel.

Each panel of our study has been approved by the Ethics and Human Subject Committees of Tongji Medical College. All participants agreed to participate in this study and signed the written informed consent.

### Copper assessment

Peripheral blood samples of participants were collected in EDTA vacuum blood collection tubes after overnight fasting. Plasma was separated from blood cells by centrifugation and stored at − 80 °C within 2 h. The concentrations of plasma Cu were measured by Agilent 7700 × inductively coupled plasma mass spectrometer (ICP-MS) after randomization, based on the methods described previously [3, 32]. A metal variability study consisted of 138 healthy participants from the DFTJ cohort was conducted previously to ensure the reproducibility of metals by comparing plasma metal concentrations at baseline and the first follow-up [32], and the intraclass correlation coefficient (ICC) for plasma Cu was 0.74 (*p* Value < 0.001), which indicates favorable reproducibility. Six samples from the DFTJ panel, twelve samples from the ACS-WH panel, two samples from the ACS-GD panel, thirty-four samples from the WHZH panel and one sample from the SY panel with non-available Cu concentrations were excluded from the study. Assessment of other covariates see Additional file 1: Method S2.

### DNA methylation and gene expression

Genome-wide DNA methylation assays for participants from the five panels and gene expression assays for the SY panel were conducted with the same protocol described previously [25]. Infinium HumanMethylation450 BeadChip (Illumina) was used to quantify DNA methylation at > 485,000 cytosine-phosphoguanine sites (CpGs) with genomic DNA extracted from whole blood of all the participants after randomization. After quality controls and normalization, 688 participants from the DFTJ panel, 102 participants from ACS-WH, 100 participants from ACS-GD, 264 participants from the WHZH panel, and 144 participants from the SY panel were retained.

HumanHT-12 version 4 Expression BeadChip (Illumina) was used to perform gene expression profiles by a commercial company (ETMD, Beijing, China) with total RNA isolated from blood leukocytes. For detailed information of experimental procedures, quality control methods, and normalization see Additional file 1: Method S3.

### Statistical analyses

#### Genome-wide analysis of DNA methylation with plasma Cu

We employed SmartSVA, a more efficient version of surrogate variable analysis (SVA), to control the potential confounding caused by cell mixtures and unknown variations in the genome-wide methylation analyses [33]. Surrogate variables (SVs) were generated from SmartSVA using variables including inverse normal transformed Cu (INT-Cu) concentration, age, sex, body mass index (BMI), smoking status, drinking status, and the proportions of major leukocytes. For the DFTJ panel and the SY panel, cellular proportions included proportions of neutrophils, lymphocytes, monocytes, eosinophils, and basophils, while for the other three panels this included proportions of neutrophils, lymphocytes, and intermediate cells (sum of monocytes, eosinophils, and basophils). To explore the associations between Cu level and CpGs, we first conducted linear regression models in five panels, separately. In each model, variables used in SmartSVA and SVs were included as covariates and INT-methylation values were included as dependent variable. Finally, we performed a fixed-effect meta-analysis to combine the association results of all the five panels, meta Z scores and *p* values were obtained by weighted sum-of-z-scores method in R 3.1.2 (R Core Team 2014). Statistical significance was determined as false discovery rate (*FDR*) < 0.05. Manhattan and QQ plots were produced using the “qqman” package to visualize the genomic distribution of significant associations. Genomic inflation lambda (λ) was calculated in R 3.1.2 (R Core Team 2014) to quantify the statistical inflation of *p* values. Regional association plot of Cu-associated CpGs was generated using “coMET” package [34], and correlation matrix of CpG sites in the region was assessed by Spearman correlation test.

#### Correlations between CpGs and gene expression level

Linear regression analysis was performed to estimate the correlations between methylation and gene expression using INT-expression values as dependent variables, and independent variables including age, sex, and methylation values without transformation. For each pair of methylation–expression probes, the significant threshold was set at *FDR* < 0.05. In addition, we searched the public database for more DNA methylation–expression associations (https://www.genenetwork.nl/biosqtlbrowser/) [35]. We used gene set enrichment analysis to examine the Kyoto Encyclopedia of Genes and Genomes (*KEGG*) pathways associated with genes annotated to the top 500 Cu-related CpG sites [36].

#### Associations of Cu-related methylation with cardiovascular risk factors

We conducted meta-analyses to investigate the association of four Cu-related CpGs with cardiovascular risk factors in the DFTJ panel, the WHZH panel, and the SY panel. In each panel, cardiovascular risk factors include BMI, systolic blood pressure (SBP), diastolic blood pressure (DBP), high-density lipoprotein cholesterol (HDL-C), low-density lipoprotein cholesterol (LDL-C), triglyceride (TG), fasting glucose (FG) and C-reactive protein (CRP), and covariates included age, sex, BMI, smoking status, and drinking status, while in the DFTJ panel we additionally adjusted for incident ACS indicator.

#### Associations of Cu-related methylation with incident ACS

To explore the association of Cu-related CpGs with incident ACS in the DFTJ panel, unconditional logistic regression models were used by including quartiles of INT-DNA methylation and continuous INT-DNA methylation values as independent variables in the model, respectively. Potential covariates in the first model including age and sex, further adjustments in the second model including BMI, smoking status, drinking status, diabetes, hypertension, and hyperlipidemia. Covariates adjusted in the second model are presented in Additional file 2: Table S1.

## Results

### Basic characteristics of the study participants

Table 1 summarizes the characteristics and plasma copper concentrations of participants by five panels. After excluding samples with failed copper detection and failed methylation quality control, a total of 1243 participants were recruited in the genome-wide meta-analysis, including 682 participants from the DFTJ cohort (male: 52.1%; mean age: 65.2 years), 90 ACS patients from Wuhan (male: 82.2%; mean age: 59.3 years), 98 ACS patients from Guangdong (male: 79.6%; mean age: 59.3 years), 230 community residents from the WHZH cohort (male: 79.1%; mean age: 54.5 years), and 143 healthy participants from Shiyan (male: 74.1%; mean age: 41.4 years). The flowchart of the study is shown in Additional file 3: Figure S1. The mean Cu concentrations were 951.5, 812.0, 897.7, 899.7, and 782.5 μg/L for the DFTJ panel, ACS-WH, ACS-GD, the WHZH panel, and the SY panel, respectively. The distribution of plasma Cu concentrations in each panel is shown in Additional file 3: Figure S2.

**Table 1:** Basic characteristics and plasma copper concentrations of the study participants

Characteristics^a^	DFTJ (*n* = 682)	ACS-WH (*n* = 90)	ACS-GD (*n* = 98)	WHZH (*n* = 230)	SY (*n* = 143)
Age, years	65.2 ± 6.3	59.3 ± 10.0	59.3 ± 11.4	54.5 ± 13.2	41.4 ± 10.2
Male, *n* (%)	355 (52.1)	74 (82.2)	78 (79.6)	182 (79.1)	106 (74.1)
Smoking status, *n* (%)					
Current smoker	160 (23.5)	33 (36.7)	41 (41.8)	95 (41.3)	45 (31.5)
Former smoker	85 (12.5)	23 (25.6)	13 (13.3)	23 (10.0)	2 (1.4)
Never smoker	437 (64.1)	34 (37.8)	44 (44.9)	112 (48.7)	96 (67.1)
Drinking status, *n* (%)					
Current drinker	164 (24.0)	21 (23.3)	20 (20.4)	65 (28.3)	54 (37.8)
Former drinker	30 (4.4)	0 (0.0)	0 (0.0)	5 (2.2)	1 (0.7)
Never drinker	488 (71.6)	69 (76.7)	78 (79.6)	160 (69.6)	88 (61.5)
BMI, kg/m^2^	24.9 ± 3.2	24.8 ± 2.8	23.0 ± 2.4	23.2 ± 2.6	24.2 ± 2.7
Systolic blood pressure, mmHg	128.9 ± 17.1	129.9 ± 20.5	134.5 ± 25.3	129.1 ± 16.7	130.7 ± 18.1
Diastolic blood pressure, mmHg	75.4 ± 10.3	81.2 ± 13.4	79.1 ± 13.8	76.9 ± 9.8	80.2 ± 12.1
Fasting glucose, mmol/L	6.1 ± 1.6	5.3 ± 0.8	6.9 ± 1.7	4.7 ± 1.3	5.5 ± 0.7
Triglyceride, mmol/L	1.5 ± 1.0	1.7 ± 1.1	1.5 ± 1.0	1.4 ± 1.0	1.4 ± 1.0
Total cholesterol, mmol/L	5.3 ± 0.9	3.9 ± 1.0	4.9 ± 1.2	5.1 ± 1.1	4.9 ± 0.8
HDL-C, mmol/L	1.4 ± 0.3	1.1 ± 0.3	1.2 ± 0.2	1.5 ± 0.4	1.4 ± 0.3
LDL-C, mmol/L	3.1 ± 0.8	2.1 ± 0.8	3.1 ± 0.9	3.0 ± 1.0	2.9 ± 0.7
Major leukocyte compositions					
Basophil proportion, %	1.3 ± 0.4	–	–	–	1.3 ± 1.2
Eosinophil proportion, %	2.2 ± 1.6	–	–	–	2.1 ± 1.6
Monocyte proportion, %	7.3 ± 1.7	–	–	–	4.9 ± 2.7
Lymphocyte proportion, %	32.8 ± 8.6	28.3 ± 9.9	19.5 ± 11.9	38.0 ± 8.2	33.6 ± 7.5
Neutrophil proportion, %	57.0 ± 9.0	62.6 ± 10.4	72.3 ± 13.4	55.3 ± 8.4	58.0 ± 8.2
Intermediate cells proportion^b^, %	10.7 ± 10.7	9.1 ± 3.1	8.2 ± 4.0	6.8 ± 3.7	8.3 ± 2.7
C-reactive protein, mg/L	2.5 ± 4.3	–	–	1.6 ± 3.7	0.8 ± 1.3
Plasma copper (μg/L)	951.5 ± 178.6	812.0 ± 183.5	897.7 ± 495.5	899.7 ± 194.3	782.5 ± 141.7

*DFTJ* the Dongfeng-Tongji Cohort, *ACS-WH* ACS patients recruited in Wuhan, *ACS-GD* ACS patients recruited in Zhuhai, *WHZH* participants from the Wuhan-Zhuhai cohort, *SY* healthy individuals from Shiyan, China

^a^The continuous variables are presented as mean ± SD. Categorical variables are presented as *n* (%)

^b^Intermediate cells consist of monocytes, eosinophils, and basophils

### Genome-wide analysis of DNA methylation and plasma copper

**Fig. 1:**
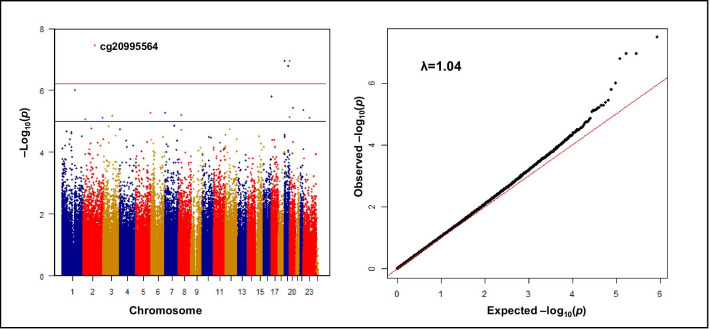
Manhattan plot and Q–Q plot for epigenome-wide association results for plasma copper concentration. The *x*-axis indicates genomic locations of the CpGs, the y-axis indicates − log10 (*p* Value) of the associations. The horizontal red line corresponds to the false discovery rate (*FDR*) = 0.05 threshold; the horizontal blue line represents suggestive association (*p* Value < 1 × 10^–5^). In the Q–Q plot, the *x*-axis shows the expected − log10 (*p* Value), whereas the *y*-axis indicates the observed − log10 (*p* Value)

The genome-wide meta-analysis identified 4 CpGs significantly associated with plasma Cu concentration with an *FDR* < 0.05 and additional 12 CpGs with *p* Values < 1 × 10^−5^ (Table 2, Fig. 1), and the results of associations between DNA methylation and plasma Cu in each panel are presented in Additional file 2: Table S2. Of the top four hits, plasma Cu was significantly associated with cg20995564 on the body of *ZEB2* gene (meta Z = − 5.52; *p* Values = 3.41 × 10^–8^), cg18608055 on the body of *SBNO2* gene (meta Z = − 5.30; *p* Values = 1.13 × 10^–7^), cg26470501 on the body of *BCL3* gene (meta Z = − 5.24; *p* Values = 1.63 × 10^–7^), and cg05825244 on the body of *EBF4* gene (meta Z = 5.30; *p* Values = 1.14 × 10^–7^).

**Table 2:** Sixteen CpGs associated with plasma copper in the genome-wide meta-analysis (*p* Value < 1 × 10^−5^)

CpG	Chr	Position	Gene	Relation to gene	Genome-wide meta-analysis of DNA methylation and plasma copper^a^		
					Meta Z	*p* Value	*FDR*
cg25112191	1	151804260	*RORC; KIAA1026; TDRKH*	1stExon, 5′UTR; < 50 kb; < 50 kb	– 4.89	9.96 × 10^–7^	0.09
cg11023668	2	25095040	*ADCY3*	Body	– 4.45	8.68 × 10^–6^	0.23
cg20995564	2	145172035	*ZEB2*	Body	– 5.52	3.41 × 10^–8^	0.01
cg09480515	2	237033375	*AGAP1; GBX2*	3′UTR; < 50 kb	– 4.47	7.75 × 10^–6^	0.22
cg21945842	3	105084992	*ALCAM*	TSS1500	– 4.51	6.48 × 10^–6^	0.22
cg24805089	5	176860947	*GRK6; DBN1; F12; PDLIM7; SLC34A1; PFN3; PRR7*	Body; < 50 kb; < 50 kb; < 50 kb; < 50 kb; < 50 kb; < 50 kb	– 4.55	5.39 × 10^–6^	0.22
cg21852842	7	1896827	*MAD1L1*	Body	– 4.55	5.47 × 10^–6^	0.22
cg18734877	8	27297419	*PTK2B; CHRNA2; EPHX2*	Body; < 50 kb; < 50 kb	– 4.52	6.33 × 10^–6^	0.22
cg07798295	16	89715839	*CHMP1A*	Body	– 4.80	1.61 × 10^–6^	0.12
cg18608055	19	1130866	*SBNO2; GPX4; HMHA1*	Body; < 50 kb; < 50 kb	– 5.30	1.13 × 10^–7^	0.02
cg26470501	19	45252955	*BCL3*	Body	– 5.24	1.63 × 10^–7^	0.02
cg24263062	20	2730191	*EBF4; CPXM1*	Body; < 50 kb	4.48	7.30 × 10^–6^	0.22
cg05825244	20	2730488	*EBF4; CPXM1*	Body; < 50 kb	5.30	1.14 × 10^–7^	0.02
cg01523712	20	43437858	*RIMS4*	Body	4.63	3.67 × 10^–6^	0.22
cg09349128	22	50327986	*ZBED4; ALG12; CRELD2; PIM3*	< 50 kb; < 50 kb; < 50 kb; < 50 kb	– 4.59	4.41 × 10^–6^	0.22
cg15376401	X	73438975	*NCRNA00182; MIR374B; MIR421*	Body; TSS1500; TSS1500	4.47	7.73 × 10^–6^	0.22

*Body* gene body, *Chr* Chromosome, *FDR* false discovery rate, *< 50 kb* within 50,000 bps of a candidate CpG site, *UTR* untranslated regions

^a^Fixed effect meta-analysis was used with a weighted sum-of-z-scores method to obtain the meta Z score and *p* Values. The significant threshold of genome-wide meta-analysis of DNA methylation and plasma copper was set at *FDR* < 0.05

More than one differentially methylated loci with *p* Values < 1 × 10^−5^ in *EBF4* gene were observed (cg05825244 at chr20:2730488 and cg24263062 at chr20:2730191). The Manhattan plot of Cu meta-analysis results surrounding the *EBF4* gene exhibited a strong comethylation structure that can be shown with strong Spearman correlation *p* values (Additional file 3: Figure S3).

### Correlations with the expression of annotated genes

We examined whether there were correlations between methylation level at the top 16 Cu-related CpGs and gene expression level in the SY panel. A total of 34 CpG-expression probe pairs annotated to 24 genes with expression rates > 30% were presented, and methylation level at 4 CpGs was significantly correlated with the expression level of annotated genes with *FDR* < 0.05 (Table 3). We additionally found two CpGs (cg26470501 and cg24263062) that were strongly associated with annotated gene expression in the public database. The genes annotated to the top 500 Cu-related CpG sites were enriched in five significant *KEGG* pathways (*FDR* < 0.01), including insulin signaling pathway, type 2 diabetes mellitus, amyotrophic lateral sclerosis, calcium signaling pathway, and purine metabolism (Additional file 2: Table S3).

**Table 3:** Methylation-gene expression correlations of the plasma copper-related CpGs (*p* Value < 1 × 10^−5^)

CpGs		Gene expression probes			Methylation–expression correlations			
CHR	CpG	Expression probe	Gene	Expression rate	Relation to gene	Effect	*p*-Value	*FDR*
1	cg25112191	ILMN_1771126	*RORC*	51.39%	1stExon;5′UTR	1.67 (3.01)	0.58	0.76
1	cg25112191	ILMN_1734366	*RORC*	84.72%	1stExon;5′UTR	1.64 (2.22)	0.74	0.84
1	cg25112191	ILMN_1680399	*KIAA1026*	65.28%	< 50 kb	− 7.51 (2.20)	9.83 × 10^–4^	3.71 × 10^–3^
1	cg25112191	ILMN_1770927	*KIAA1026*	87.50%	< 50 kb	− 7.43 (2.05)	4.33 × 10^–4^	1.84 × 10^–3^
1	cg25112191	ILMN_1798458	*KIAA1026*	95.83%	< 50 kb	− 7.19 (1.98)	3.91 × 10^–4^	2.22 × 10^–3^
1	cg25112191	ILMN_1792726	*TDRKH*	36.81%	< 50 kb	1.15 (2.76)	0.68	0.82
2	cg11023668	ILMN_1676893	*ADCY3*	100.00%	Body	− 0.51 (0.92)	0.58	0.73
2	cg20995564	ILMN_1688698	*ZEB2*	100.00%	Body	1.39 (1.26)	0.27	0.46
3	cg21945842	ILMN_1670870	*ALCAM*	100.00%	TSS1500	3.25 (3.58)	0.37	0.57
5	cg24805089	ILMN_2357015	*GRK6*	100.00%	Body	− 2.42 (4.28)	0.57	0.78
5	cg24805089	ILMN_1681802	*GRK6*	100.00%	Body	0.68 (4.37)	0.88	0.97
7	cg21852842	ILMN_2358069	*MAD1L1*	100.00%	Body	3.14 (1.73)	0.07	0.19
8	cg18734877	ILMN_1732318	*PTK2B*	100.00%	Body	5.44 (2.21)	0.01	0.05
8	cg18734877	ILMN_2330966	*PTK2B*	100.00%	Body	3.51 (2.18)	0.11	0.23
8	cg18734877	ILMN_1698849	*CHRNA2*	82.64%	< 50 kb	0.15 (2.53)	0.95	0.98
8	cg18734877	ILMN_1709237	*EPHX2*	100.00%	< 50 kb	− 2.03 (2.19)	0.35	0.57
16	cg07798295	ILMN_1709439	*CHMP1A*	100.00%	Body	− 2.63 (3.67)	0.47	0.67
19	cg18608055	ILMN_1808811	*SBNO2*	100.00%	Body	7.28 (1.81)	9.53 × 10^–5^	6.48 × 10^–4^
19	cg18608055	ILMN_1734353	*GPX4*	100.00%	< 50 kb	− 10.28 (1.78)	4.48 × 10^–8^	7.62 × 10^–7^
19	cg18608055	ILMN_2378952	*GPX4*	100.00%	< 50 kb	− 10.57 (1.77)	1.84 × 10^–8^	6.26 × 10^–7^
19	cg18608055	ILMN_1811392	*HMHA1*	100.00%	< 50 kb	9.28 (1.79)	7.40 × 10^–7^	8.39 × 10^–6^
19	cg18608055	ILMN_3239240	*POLR2E*	100.00%	< 50 kb	0.78 (1.97)	0.69	0.81
19	cg26470501^a^	ILMN_1710514	*BCL3*	100.00%	Body	− 3.77 (2.55)	0.14	0.27
20	cg24263062^a^	ILMN_3261292	*EBF4*	90.97%	Body	2.01 (1.33)	0.14	0.27
20	cg24263062	ILMN_1712046	*CPXM1*	66.67%	< 50 kb	2.57 (1.49)	0.09	0.21
20	cg05825244^a^	ILMN_3261292	*EBF4*	90.97%	Body	1.25 (0.73)	0.09	0.21
20	cg05825244	ILMN_1712046	*CPXM1*	66.67%	< 50 kb	2.06 (0.82)	0.01	0.04
20	cg01523712	ILMN_1746031	*RIMS4*	59.44%	Body	1.80 (2.39)	0.45	0.67
22	cg09349128	ILMN_1782129	*ZBED4*	100.00%	< 50 kb	10.42 (2.88)	4.20 × 10^–4^	2.04 × 10^–3^
22	cg09349128	ILMN_1743995	*ALG12*	97.22%	< 50 kb	− 0.14 (3.00)	0.96	0.96
22	cg09349128	ILMN_1748707	*CRELD2*	100.00%	< 50 kb	− 0.20 (2.97)	0.95	1.00
22	cg09349128	ILMN_1672034	*PIM3*	48.95%	< 50 kb	− 5.22 (3.86)	0.18	0.33
22	cg09349128	ILMN_1707748	*PIM3*	100.00%	< 50 kb	− 12.08 (2.76)	2.34 × 10^–5^	1.99 × 10^–4^
22	cg09349128	ILMN_1789781	*PIM3*	88.11%	< 50 kb	− 7.22 (3.04)	0.02	0.06

Methylation–expression correlations were calculated using linear regressions in which inverse normal transformed expression values were regressed on original methylation values adjusted for age and gender. The significant threshold of methylation–expression associations was set at *FDR* < 0.05

^a^DNA methylation level of cg26470501 was reported to associated with expression of *BCL3* gene; DNA methylation level of cg24263062 and cg05825244 was reported to positively associated with *EBF4* gene in public database (*FDR* < 0.05) (https://www.genenetwork.nl/biosqtlbrowser/)

### Relations to the cardiovascular risk factors

We tested the associations of four Cu-related CpGs (*FDR* < 0.05) with cardiovascular risk factors in participants who were not currently ACS patients. Methylation level of cg05825244 presented significant correlations with serum HDL-C level (*p* Value = 0.005) and CRP level (*p* Value = 0.03), we also observed a significant correlation between methylation level of cg26470501 and serum CRP level (*p* Value = 0.003, Additional file 2: Table S4). We did not find other risk factors associated with Cu-related CpGs.

### Associations of Cu-related CpGs with incident ACS

We examined the associations of Cu-related CpGs with incident ACS in the nested case-control participants within DFTJ cohort and observed that higher methylation level at cg05825244 locus was associated with an increased risk of ACS: The OR (95% CI) compared the fourth quartile with the first quartile was 1.78 (1.07, 2.98; *p*-Trend = 0.04), and for linear model, the OR (95% CI) was 1.23 (1.02, 1.48; *p*-Linearity = 0.03) according to per INT-DNA methylation values increase (Table 4).

**Table 4:** Adjusted odds ratios for the incident ACS according to quartiles of DNA methylation level

	Quartiles of methylation level^a^				*p*-Trend^b^	Linear model^c^	*p-*Linearity^d^
	Q1	Q2	Q3	Q4			
cg20995564	< 0.3834	0.3834–0.4141	0.4141–0.4473	≥ 0.4473			
* n* (case/control)	85/85	88/82	82/89	86/85			
Model 1^e^	1.00 (ref)	1.07 (0.70, 1.64)	0.92 (0.60, 1.41)	1.01 (0.66, 1.54)	0.89	1.02 (0.88, 1.19)	0.78
Model 2^f^	1.00 (ref)	0.96 (0.59, 1.58)	0.92 (0.56, 1.51)	0.77 (0.47, 1.27)	0.30	0.95 (0.80, 1.13)	0.55
cg18608055	< 0.4760	0.4760–0.5140	0.5140–0.5560	≥ 0.5560			
* n* (case/control)	75/95	92/79	80/90	94/77			
Model 1^e^	1.00 (ref)	1.48 (0.96, 2.27)	1.13 (0.73, 1.73)	1.55 (1.01, 2.38)	0.12	1.09 (0.94, 1.27)	0.26
Model 2^f^	1.00 (ref)	1.53 (0.93, 2.52)	1.06 (0.64, 1.75)	1.23 (0.75, 2.03)	0.74	1.00 (0.84, 1.19)	0.98
cg26470501	< 0.3905	0.3905–0.4170	0.4170–0.4395	≥ 0.4395			
*n* (case/control)	92/79	82/88	80/91	87/83			
Model 1^e^	1.00 (ref)	0.80 (0.52, 1.22)	0.75 (0.49, 1.15)	0.89 (0.57, 1.37)	0.51	0.90 (0.77, 1.05)	0.19
Model 2^f^	1.00 (ref)	0.74 (0.45, 1.23)	0.86 (0.52, 1.43)	1.06 (0.63, 1.79)	0.75	0.96 (0.79, 1.15)	0.63
cg05825244	< 0.4458	0.4458–0.5636	0.5636–0.6648	≥ 0.6648			
*n* (case/control)	74/97	89/81	86/85	92/78			
Model 1^e^	1.00 (ref)	1.45 (0.95, 2.23)	1.34 (0.87, 2.06)	1.58 (1.02, 2.44)	0.06	1.17 (1.00, 1.37)	0.05
Model 2^f^	1.00 (ref)	1.45 (0.88, 2.39)	1.40 (0.85, 2.31)	1.78 (1.07, 2.98)	0.04	1.23 (1.02, 1.48)	0.03

^a^DNA methylation level of CpGs was presented as relative level after normalization

^b^*P* for trend across quartiles of CpGs were obtained by including the median of each quartile as a continuous variable in logistic regression models

^c^Odds ratios (95% CI) for incident ACS correspond to one-unit increase in inverse normal transformation of methylation level

^d^*P* for linearity were obtained by including inverse normal transformation methylation values as continuous variable in linear regression models

^e^Model 1 was adjusted for age and sex in the unconditional logistic regression model

^f^Model 2 was additionally adjusted for body mass index, smoking status, drinking status, hypertension, hyperlipidemia, and diabetes in the unconditional logistic regression model

## Discussion

We integrated genome-wide DNA methylation and gene expression profiles in leukocyte of 1243 Chinese individuals to examine the gene-specific DNA methylation changes associated with Cu concentration and explore the functional consequences by assessing gene expression and clinical outcomes associated with identified CpGs. We observed four novel methylation loci in *ZEB2*, *SBNO2*, *BCL3*, and *EBF4* that were strongly associated with Cu exposure (*FDR* < 0.05) and additional 12 CpGs with suggestive associations (*p* Value < 1 × 10^−5^). Three of the four identified CpGs showed lower methylation level in association with higher Cu exposure. In addition, we observed that several of the differentially methylated loci related to plasma Cu were associated with corresponding gene expression level, indicating that copper may regulate gene expression by altering DNA methylation level. We also demonstrated that the DNA methylation level at cg05825244 was associated with lower HDL-C level and higher CRP level. Most notably, we observed that methylation alteration of cg05825244 was statistically significantly associated with increased risk of ACS.

Copper is an essential but toxic trace element for the human body and the major dietary sources of copper are legumes, potato, nuts, and beef [37]. Previous metal variability study has shown that plasma copper is a reliable biomarker reflecting chronic copper exposure with an ICC of 0.74 over five years [32]. A recent meta-analysis revealed that exposure to Cu was associated with increased risk of CVD and CHD by comparing top versus bottom thirds of baseline level [4]. A prospective study consisting of 1666 men in Eastern Finland demonstrated that high copper status was an independent risk factor for acute myocardial infarction (AMI), with an average copper level of 1110 μg/L in participants [6]. Xiao et al. [3] reported that elevated plasma copper concentrations were associated with a higher risk of stroke in a linear dose–response manner: The odds ratio according to per interquartile range increase of Cu was 1.29 (95% CI 1.13, 1.46), and the median Cu concentration in the control group of this study was 963.4 μg/L. Our study participants had a wide scope of Cu concentrations, ranging from 200 to 1600 μg/L. The observed preference for Cu-associated hypomethylation was consistent with the findings of previous study that higher Cu was associated with a lower global DNA methylation profile of leukocytes [22]. Kennedy et al. [23] also observed that hypomethylation is more common than hypermethylation among top CpGs associated with copper concentration. They also suggested that copper-associated DNA methylation patterns in placentae may mediate normal placenta and fetal development. However, they did not find a statistically significant association between copper status and methylation status at any single CpG. The DNA methylation alterations identified in the current study may provide novel insights into the biological function of copper and the association of Cu with CVD.

Higher arsenic exposure was associated with increased methylation level at cg05825244 (chr20: 2730488) and cg24263062 (chr20: 2730191), both located in the gene body of *EBF Family Member 4* (*EBF4*). These two loci were found to be strongly associated with increased gene expression of *EBF4* in public database [35], and we also observed that cg05825244 was moderately associated with increased expression of its nearby gene *CPXM1* in our gene expression profile data. We also found several other methylation loci on *EBF4* gene were associated with Cu concentrations. *EBF4* encodes transcription factor COE4, which has an essential regulatory function in neural development and B-cell maturation [38]. Interestingly, it has been suggested that EBF4 had a protein–protein interaction with APP, which has a Cu-binding domain that can reduce Cu^2+^ to Cu^+^ with oxidative damage [39]. We observed that increased methylation level of cg05825244 related to higher Cu exposure was associated with lower HDL-C level and increased risk of ACS. Exposure to excess Cu has been demonstrated to initiate oxidative damage in several in vivo and in vitro studies, which may induce lipid peroxidation, endothelial dysfunction, and vascular injury [8, 9]. Our results suggest that copper-associated DNA methylation alterations may mediate abnormal lipid metabolism and thus participate in the development of ACS. Nevertheless, the results from the current study do not infer causal directionality. It is necessary to conduct more research to explore the biological implications of these findings (Additional file 4).

The top hit from our study is cg20995564 (chr2: 145172035), located in the body of *ZEB2* gene. The protein encoded by *ZEB2* is a member of the Zfh1 family of two-handed zinc finger/homeodomain proteins. The related pathways of *ZEB2* are TGF-beta Receptor Signaling and MicroRNAs in cancer. *ZEB2* has been implicated to be associated with several diseases including Mowat-Wilson syndrome and esophageal cancer [40]. Mutations in this gene have been associated with total cholesterol (TC) and LDL-C [41]. Furthermore, we found the methylation level of cg20995564 have been identified in an EWAS of CRP [42]. Plasma Cu has shown a strong positive correlation with CRP concentrations and inflammation [43]. It suggests that increased inflammation level associated with Cu could be mediated by methylation, but more research is warranted to verify the specific mechanism.

Higher Cu exposure was associated with decreased methylation level at the cg18608055 locus (chr19: 1130866) located in the body of *SBNO2* gene and cg26470501 locus located in the body of *BCL3* gene (chr19: 45252955). These two loci were strongly associated with increased gene expression of *SBNO2* and decreased gene expression of *BCL3*, respectively. Of interest, the decreased methylation level of these two loci was also strongly associated with higher CRP concentrations and higher risk of inflammatory bowel disease (IBD) in previous EWAS studies [42, 44]. Consistently, we verified that the methylation alteration at cg26470501 was associated with CRP level. *SBNO4* encodes the strawberry notch homolog 2 protein, which is involved in the transcriptional corepression of NF-κB in macrophages, as well as proinflammatory cascade [45]. *BCL3* is a proto-oncogene candidate gene with the function of transcriptional co-activation that activates through its association with NF-κB homodimers. The expression of *BCL3* gene can be induced by NF-κB, which forms a part of the autoregulatory loop that controls the nuclear residence of p50 NF-κB [46]. The mutations in *BCL3* have been associated with HDL-C level, fasting insulin level and Alzheimer's disease (AD) [47]. Elevated Cu has been observed in cancer, IBD and AD patients in previous studies [48–50]. Taken together, these findings indicate that DNA methylation has the potential to explain the association of Cu with lipid metabolism, inflammatory response, and Cu-related diseases.

Our top 500 sites included genes that were significantly enriched for insulin signaling pathway and type 2 diabetes mellitus pathway. Exposure to higher Cu level has primarily been reported to relate to a higher risk of diabetes [51]. Our findings provide evidence that these biological pathways of interest in humans may be perturbed by Cu-associated differential DNA methylation alterations and further lead to diseases.

To the best of our knowledge, this was the first study to identify the significant CpGs associated with plasma Cu at genome-wide DNA methylation level. The major strengths of the present study are the relatively large sample size of the study and the availability of genome-wide methylation data. The combination of the epigenome-wide methylation data with genome-wide expression data as well as gene enrichment analyses allowed us to evaluate potentially functional gene regulation associated with the differentially methylated loci. In addition, the prospective design of nested case-control study for ACS gave us the chance to investigate the associations of Cu-related CpGs with future ACS. Further integration of these findings with clinical outcome data suggested for the first time that DNA methylation had a potential role as an intermediate biomarker for in the complex interplay between cooper, lipid metabolism, and ACS risk.

This study also has several potential limitations. First, this was an observational study measuring plasma Cu concentrations and DNA methylation level at the same time point. Therefore, the causality between the Cu concentrations and DNA methylation variations could not be determined. Further relevant longitudinal study and large Mendelian randomization studies are needed. Second, although we used the SmartSVA method and adjusted for major leukocyte compositions to control for the confounding effects of cell composition batch, potential cofounding still exists. Future studies of DNA methylation in specific cell type for different tissues may be able to solve this limitation. Third, although we adjusted for several confounders and used SmartSVA to remove significant unknown confounding effects in our study, we cannot rule out all the unmeasured or residual confounding. Fourth, the participants in our study are all Chinese adults, we did not validate the identified methylation signals in other populations; therefore, the generalization to other populations is limited. Fifth, the assay that we used to measure plasma copper did not distinguish between protein-bound copper and free copper forms. In the human body, 85% to 95% of the blood copper is covalently bound to the ceruloplasmin molecule, and another 5% to 15% is loosely bound to albumin and small molecules in the blood in the form of free copper [52]. Free copper is a harmful form that can produce reactive oxygen species and cause cell damage [53]. More DNA methylation studies focusing on free copper are needed in the future. Finally, although we have identified novel methylation loci for plasma copper, we were unable to investigate the exact functional mechanisms behind the observed methylation changes associated with Cu exposure, and the small number of identified differentially methylated loci may limit our clinical relevance. Despite these limitations, we believed that our findings provided evidence for potential mechanisms involving DNA methylation alterations that may partly explain the association of copper with ACS. Further studies are warranted to explore the biological implications of these findings.

## Conclusion

In summary, we identified four novel DNA methylation sites related to Cu exposure, three of which were associated with corresponding gene expression level. In addition, we observed that Cu-related DNA methylation alteration at cg05825244 was associated with increased risk of ACS, which may be attributed to abnormal lipid metabolism and inflammation. The findings from the current study may provide new insights regarding the epigenetic regulation mechanisms between copper and cardiovascular diseases and suggest potential targets for intervention or prevention of copper-related clinical consequences.

## Supplementary information

**Additional file 1.** Method S1: Details of the study population. Method S2: Covariate assessment. Method S3: Detailed processing methods for DNA methylation and gene expression.

**Additional file 2.** Table S1: Covariates used in associations of four copper-associated CpGs with incident ACS in the DFTJ panel. Table S2: Results from panel-specific analyses of plasma copper-associated DNA methylation, for probes with meta-analysis p-Value <1×10^−5^, sorted by chromosome. Table S3: Significantly enriched *KEGG* pathways (*FDR* <0.05) associated with genes annotated to the top 500 CpG sites. Table S4: Meta-analysis of associations between four plasma copper-related CpGs (*FDR* <0.05) with major cardiovascular risk factors.

**Additional file 3.** Figure S1: The flowchart of the study. Figure S2: Distribution of plasma copper concentrations in five panels. Figure S3: Regional association plot of Cu meta-analysis results within and surrounding the *EBF4* gene.

**Additional file 4.** This file presents a complete set of the meta-analysis results of the EWAS for plasma copper in this study.

## Abbreviations

ACS: Acute coronary syndrome
AD: Alzheimer’s disease
AMI: Acute myocardial infarction
BMI: Body mass index
CpGs: Cytosine-phosphoguanines
CRP: C-reactive protein
Cu: Copper
CVD: Cardiovascular disease
DBP: Diastolic blood pressure
DFTJ cohort: Dongfeng-Tongji cohort
DMC: Dongfeng Motor Corporation
DMRs: Differentially methylated regions
EWAS: Epigenome-wide association study
*FDR*: False discovery rate
FG: Fasting glucose
HDL-C: High-density lipoprotein cholesterol
IBD: Inflammatory bowel disease
ICC: Intraclass correlation coefficient
ICP-MS: Inductively coupled plasma mass spectrometer
INT: Inverse normal transformed
*KEGG*: Kyoto Encyclopedia of Genes and Genomes
LDL-C: Low-density lipoprotein cholesterol
NSTEMI: Non-ST-segment elevation myocardial infarction
SBP: Systolic blood pressure
SVs: Surrogate variables
SY: Shiyan
TC: Total cholesterol
TG: Triglyceride
UA: Unstable angina
WHZH: Wuhan-Zhuhai
