# Supplementary material for: Profile of copper-associated DNA methylation and its association with incident acute coronary syndrome
Source: Clin Epigenetics. 2021 Jan 27;13:19. doi: 10.1186/s13148-021-01004-w (PMC7839231; doi:10.1186/s13148-021-01004-w)
Supplement: Supplementary file 1 — Additional file 1. Method S1: Details of the study population. Method S2: Covariate assessment. Method S3: Detailed processing methods for DNA methylation and gene expression. [file 13148_2021_1004_MOESM1_ESM.docx]

**Additional file 1.**

**Profile of copper-associated DNA methylation and its association with incident acute coronary syndrome**

Pinpin Long^†^, Qiuhong Wang^†^, Yizhi Zhang, Xiaoyan Zhu, Kuai Yu, Haijing Jiang, Xuezhen Liu, Min Zhou, Yu Yuan, Kang Liu, Jing Jiang, Xiaomin Zhang, Meian He, Huan Guo, Weihong Chen, Jing Yuan, Longxian Cheng, Liming Liang, and Tangchun Wu^*^

**Method S1.** Details of the study population.

**Method S2.** Covariate assessment.

**Method S3.** Detailed processing methods for DNA methylation and gene expression.

**Method S1**. Details of the study population.

***The Dongfeng-Tongji (DFTJ) panel for incident ACS.*** The DFTJ cohort is a prospective cohort based on retired workers from Dongfeng Motor Corporation (DMC) in Shiyan City, Hubei province, China. The DFTJ cohort recruited 27,009 participants from September 2008 to June 2010. Questionnaires, medical examinations, and fasting blood samples were conducted at baseline [1]. At the first follow-up, 96.2% of the participants repeated the questionnaire interview, medical examinations, and blood collection. At the same time, the incidents of CVD, diabetes, and cancer were registered up to 31 December 2013 [2].

The incident ACS cases and matched controls were selected among participants who a) donated blood at baseline; b) participated in the follow-up or died during follow-up; c) did not report coronary heart disease (CHD), stroke or cancer at baseline; and d) did not have abnormal baseline electrocardiograph (ECG). A total of 1,146 incident ACS cases were determined till 31 December 2013. We conducted a nested case-control study by randomly selected 344 incident ACS cases and 344 controls matched for age (±3 y), sex, and sampling time at baseline (±40 d).

***Two prevalent ACS patient panels.*** A two-stage genome-wide methylation association analysis was performed to explore prevalent ACS related DNA methylation alterations in Wuhan and Zhuhai, China, respectively. The analysis selected 103 ACS patients in Wuhan (abbreviated as ACS-WH) and 103 ACS patients in Zhuhai (abbreviated as ACS-GD) who a) donated blood at the earliest time of admission before any clinic treatment; b) were clinically diagnosed with acute myocardial infarction (AMI) or UA; c) were free of other cardiovascular diseases, chronic obstructive pulmonary disease (COPD), diabetes, acute or chronic infectious diseases, severe liver or kidney diseases and cancers [3, 4]. We recruited controls of the prevalent ACS patients from the Wuhan-Zhuhai (WHZH) cohort, a cohort conducted in communities of two cities named Wuhan and Zhuhai in China (abbreviated as the WHZH panel). Established in 2011, this cohort recruited 3,053 residents from Wuhan and 1,759 residents from Zhuhai, using a stratified, cluster sampling approach. Participants aged from 18 to 80, free of severe illnesses, and had lived in the sampling sites for more than 5 years [9]. 180 Wuhan residents and 103 Zhuhai residents were selected following the criteria below: a) donated blood and urine samples; b) did not report any acute or chronic diseases; c) did not report infectious diseases or use medications within 4 weeks before blood sampling.

***The Shiyan (SY) panel.*** To investigate whether DNA methylation was associated with gene expression, we recruited 144 individuals who had regular physical examinations at the Health Examination Center of Dongfeng Central Hospital (Dongfeng Motor Corporation and Hubei University of Medicine) in Shiyan, between April and May 2015. Participants enrolled in the study a) were aged from 20 to 70; b) donated both blood and urine samples; c) did not report any chronic diseases; d) did not report fever or infectious conditions in two weeks before baseline examination and e) denied the use of medications in four weeks before the baseline examination.

**Method S2.** Covariate assessment.

Information on age, sex, height, weight, smoking status (never, former, current) and drinking status (never, former, current) were collected through questionnaires at baseline of each panel. Body mass index (BMI) was calculated by formula BMI=weight (kg)/height (m^2^). Current smokers were defined as smoking at least 1 cigarette daily for more than half a year. Current drinkers were defined as drinking at least once a week for more than half a year [9]. Data on fasting glucose (FG), blood pressure (BP), lipid profiles, and proportions of major leukocytes in each panel, and serum C-reactive protein (CRP) in the DFTJ panel were measured using fasting blood collected from physical examination at baseline. In the DFTJ panel, diabetes was defined as FG ≥7.0 mmol/L, or have self-reported physician-diagnosed diabetes or currently use pharmacological treatment with oral agents or insulin. Hypertension was defined as systolic blood pressure (SBP)/diastolic blood pressure (DBP) ≥140/90 mmHg, or self-reported physician-diagnosed hypertension or currently use anti-hypertension drugs. Hyperlipidemia was defined as total cholesterol (TC) ≥6.22 mmol/L, or triglyceride (TG) ≥2.26 mmol/L, or HDL-C <1.04 mmol/L or low-density lipoprotein cholesterol (LDL-C) ≥4.14 mmol/L, or self-reported physician-diagnosed hyperlipidemia or currently use lipid-lowering drugs [9].

**Method S3.** Detailed processing methods for DNA methylation and gene expression.

Genome-wide DNA methylation assays for participants from the five panels were conducted with the same protocol described previously [9]. Brieﬂy, we used the BioTeke Whole Blood DNA Extraction Kit (BioTeke) to extract genomic DNA and stored them at –80°C. A total of 1,000 ng genomic DNA of each sample was bisulfite converted with Zymo EZ DNA Methylation kit (Zymo Research) and diluted to 10μL with a concentration of 60 ng/μL. After randomization, Inﬁnium HumanMethylation450 BeadChip (Illumina) was used to quantify DNA methylation at >485,000 cytosine-phosphoguanine (CpG) sites with 4 μL bisulfite converted samples. IDat ﬁles generated from processed beadchips through the iScan (Illumina) system were estimated using the “minﬁ” package of R (version 3.3.1, R Core Team) [9]. Probes were filtered if they met the following criteria: a) single nucleotide polymorphisms (SNPs) on the Methylation450k Beadchips; b) missing rate >20% (a probe was defined as missing when detection *p*-Value >0.01 or bead counts <3); c) potentially contained or extended on SNPs with minor allele frequency (MAF) >0.05 in the 1000 Genomes Project 20110521 release for ASN population, or probes possibly cross-hybridized to other genomic locations (41,296 probes). Samples were excluded if they met the following criteria: a) outliers detected in multidimensional scaling (MDS) analysis; b) mix-up samples; c) missing rate >5% across probes; d) failed quality controls of sex discrepancies based on genome-wide association study (GWAS) data [3]. After quality control, raw methylation data were normalized using the dasen method in the wateRmelon package [4].

gene expression assays for the SY panel were described previously [9]. Brieﬂy, total RNA was isolated from blood leukocytes within two hours after blood sampling, using TRIzol® LS solution (Invitrogen) according to the manufacturer’s instructions. HumanHT-12 version 4 Expression BeadChip (Illumina) was used to perform gene expression profiles by a commercial company (ETMD, Beijing, China). Raw expression data were then obtained using GenomeStudio (Illumina) and quantile-quantile normalized using the “beadarray” package [9] in R 3.1.2 (R Core Team 2014).

**References**

1. Wang F, Zhu J, Yao P, Li X, He M, Liu Y, et al. Cohort Profile: the Dongfeng-Tongji cohort study of retired workers. Int J Epidemiol. 2013;42(3):731-740. https://doi.org/10.1093/ije/dys053.

2. Yuan Y, Xiao Y, Feng W, Liu Y, Yu Y, Zhou L, et al. Plasma metal concentrations and incident coronary heart disease in Chinese adults: The Dongfeng-Tongji cohort. Environ Health Perspect. 2017;125(10):107007. https://doi.org/10.1289/EHP1521.

3. Li J, Zhu X, Yu K, Jiang H, Zhang Y, Deng S, et al. Genome-wide analysis of DNA methylation and acute coronary syndrome. Circ Res. 2017;120(11):1754-1767. https://doi.org/10.1161/CIRCRESAHA.116.310324.

4. Pidsley R, Y Wong CC, Volta M, Lunnon K, Mill J, Schalkwyk LC. A data-driven approach to preprocessing Illumina 450K methylation array data. BMC genomics. 2013;14:293. https://doi.org/10.1186/1471-2164-14-293.

5. Zhu X, Li J, Deng S, Yu K, Liu X, Deng Q, et al. Genome-wide analysis of DNA methylation and cigarette smoking in a Chinese population. Environ Health Perspect. 2016;124(7):966-973. https://doi.org/10.1289/ehp.1509834.

6. Song Y, Hou J, Huang X, Zhang X, Tan A, Rong Y, et al. The Wuhan-Zhuhai (WHZH) cohort study of environmental air particulate matter and the pathogenesis of cardiopulmonary diseases: study design, methods and baseline characteristics of the cohort. BMC Public Health. 2014;14:994. https://doi.org/10.1186/1471-2458-14-994.

7. Zhou L, Yu K, Yang L, Wang H, Xiao Y, Qiu G, et al. Sleep duration, midday napping, and sleep quality and incident stroke: The Dongfeng-Tongji cohort. Neurology. 2020;94(4):e345-e356, https://doi.org/10.1212/WNL.0000000000008739.

8. Aryee MJ, Jaffe AE, Corrada-Bravo H, Ladd-Acosta C, Feinberg AP, Hansen KD, et al. Minfi: A flexible and comprehensive Bioconductor package for the analysis of Infinium DNA methylation microarrays. Bioinformatics. 2014;30(10):1363-1369. https://doi.org/10.1093/bioinformatics/btu049.

9. Dunning MJ, Smith ML, Ritchie ME, Tavaré S. Beadarray: R classes and methods for Illumina bead-based data. Bioinformatics. 2014;23(16):2183-2184. https://doi.org/10.1093/bioinformatics/btm311.
