# Supplementary material for: Profile of copper-associated DNA methylation and its association with incident acute coronary syndrome
Source: Clin Epigenetics. 2021 Jan 27;13:19. doi: 10.1186/s13148-021-01004-w (PMC7839231; doi:10.1186/s13148-021-01004-w)
Supplement: Supplementary file 2 — Additional file 2. Table S1: Covariates used in associations of four copper-associated CpGs with incident ACS in the DFTJ panel. Table S2: Results from panel-specific analyses of plasma copper-associated DNA methylation, for probes with meta-analysis p-Value <1×10^−5^, sorted by chromosome. Table S3: Significantly enriched KEGG pathways (FDR <0.05) associated with genes annotated to the top 500 CpG sites. Table S4: Meta-analysis of associations between four plasma copper-related CpGs (FDR <0.05) with major cardiovascular risk factors. [file 13148_2021_1004_MOESM2_ESM.docx]

**Additional file 2.**

**Profile of copper-associated DNA methylation and its association with incident acute coronary syndrome**

Pinpin Long^†^, Qiuhong Wang^†^, Yizhi Zhang, Xiaoyan Zhu, Kuai Yu, Haijing Jiang, Xuezhen Liu, Min Zhou, Yu Yuan, Kang Liu, Jing Jiang, Xiaomin Zhang, Meian He, Huan Guo, Weihong Chen, Jing Yuan, Longxian Cheng, Liming Liang, and Tangchun Wu^*^

**Table S1.** Covariates used in associations of four copper-associated CpGs with incident ACS in the DFTJ panel.

**Table S2.** Results from panel-specific analyses of plasma copper-associated DNA methylation, for probes with meta-analysis *p*-Value <1×10^−5^, sorted by chromosome.

**Table S3.** Significantly enriched *KEGG* pathways (*FDR* <0.05) associated with genes annotated to the top 500 CpG sites.

**Table S4.** Meta-analysis of associations between four plasma copper-related CpGs (*FDR* <0.05) with major cardiovascular risk factors.

| Table S1. Covariates used in associations of four copper-associated CpGs with incident ACS in the DFTJ panel. | | | |
| --- | --- | --- | --- |
| Characteristics*^a^* | Controls (n=341) | Cases (n=341) | *P* value*^b^* |
| Age, years | 65.2 ± 6.3 | 65.1 ± 6.4 | 0.81 |
| Male, *n* (%) | 163 (47.8) | 164 (48.1) | - |
| BMI, kg/m^2^ | 24.7 ± 3.0 | 25.0 ± 3.3 | 0.15 |
| Smoking status, *n* (%) |  |  |  |
| Current smoker | 75 (22.0) | 85 (24.9) | 0.49 |
| Former smoker | 40 (11.7) | 45 (13.2) |  |
| Never smoker | 226 (66.3) | 211 (61.9) |  |
| Drinking status, *n* (%) |  |  |  |
| Current drinker | 96 (28.2) | 68 (19.9) | 0.003 |
| Former drinker | 8 (2.3) | 22 (6.5) |  |
| Never drinker | 237 (69.5) | 251 (73.6) |  |
| Hypertension | 0.25 (0.43) | 0.67 (0.47) | <0.001 |
| Hyperlipidemia | 0.35 (0.48) | 0.59 (0.49) | <0.001 |
| Diabetes | 0.06 (0.23) | 0.27 (0.44) | <0.001 |
| Note: ACS, acute coronary syndrome; BMI, body mass index; DFTJ, the Dongfeng-Tongji Cohort. | | | |
| *^a^*The continuous variables are presented as mean ± SD. Categorical variables are presented as *n* (%). | | | |
| *^b^P* values were derived from Student’s t test or the Mann-Whitney U test for continuous variables, and Chi-square test for the category variables. | | | |

| Table S2. Results from panel-specific analyses of plasma copper-associated DNA methylation, for probes with meta-analysis *p*-Value <1×10^−5^, sorted by chromosome. | | | | | | | | | | | | | | | |
| --- | --- | --- | --- | --- | --- | --- | --- | --- | --- | --- | --- | --- | --- | --- | --- |
|  |  | DFTJ (n=682) | |  | ACS-WH (n=90) | |  | ACS-GD (n=98) | |  | WHZH (n=230) | |  | SY (n=143) | |
| CpG | Chr | Effect (SE)*^a^* | *p*-Value |  | Effect (SE)*^a^* | *p*-Value |  | Effect (SE)*^a^* | *p*-Value |  | Effect (SE)*^a^* | *p*-Value |  | Effect (SE)*^a^* | *p*-Value |
| cg25112191 | 1 | –0.152 (0.036) | 2.24E–05 |  | –0.218 (0.106) | 0.044 |  | 0.011 (0.096) | 0.909 |  | –0.039 (0.054) | 0.474 |  | –0.221 (0.078) | 5.92E–03 |
| cg20995564 | 2 | –0.167 (0.038) | 1.29E–05 |  | –0.151 (0.076) | 0.052 |  | 0.009 (0.094) | 0.920 |  | –0.078 (0.045) | 0.085 |  | –0.297 (0.093) | 1.89E–03 |
| cg09480515 | 2 | –0.140 (0.040) | 4.38E–04 |  | –0.331 (0.120) | 7.65E–03 |  | –0.163 (0.122) | 0.185 |  | –0.057 (0.071) | 0.426 |  | –0.133 (0.103) | 0.199 |
| cg11023668 | 2 | –0.157 (0.039) | 6.36E–05 |  | –0.166 (0.128) | 0.197 |  | –0.262 (0.124) | 0.037 |  | –0.051 (0.070) | 0.465 |  | –0.073 (0.103) | 0.481 |
| cg21945842 | 3 | –0.112 (0.037) | 2.98E–03 |  | –0.221 (0.080) | 7.85E–03 |  | –0.119 (0.081) | 0.146 |  | –0.080 (0.046) | 0.087 |  | –0.145 (0.108) | 0.183 |
| cg24805089 | 5 | –0.104 (0.033) | 2.00E–03 |  | –0.218 (0.089) | 0.016 |  | –0.144 (0.078) | 0.070 |  | –0.063 (0.059) | 0.286 |  | –0.175 (0.091) | 0.057 |
| cg21852842 | 7 | –0.149 (0.037) | 6.26E–05 |  | 0.018 (0.110) | 0.873 |  | –0.140 (0.094) | 0.140 |  | –0.140 (0.062) | 0.026 |  | –0.069 (0.092) | 0.452 |
| cg18734877 | 8 | –0.145 (0.036) | 6.69E–05 |  | –0.125 (0.111) | 0.263 |  | –0.102 (0.092) | 0.272 |  | –0.096 (0.066) | 0.151 |  | –0.093 (0.094) | 0.324 |
| cg07798295 | 16 | –0.129 (0.033) | 1.21E–04 |  | –0.026 (0.088) | 0.769 |  | –0.031 (0.088) | 0.728 |  | –0.094 (0.059) | 0.113 |  | –0.330 (0.099) | 1.25E–03 |
| cg18608055 | 19 | –0.096 (0.024) | 8.67E–05 |  | –0.107 (0.074) | 0.156 |  | –0.077 (0.063) | 0.224 |  | –0.084 (0.035) | 0.019 |  | –0.095 (0.048) | 0.051 |
| cg26470501 | 19 | –0.160 (0.037) | 1.38E–05 |  | –0.065 (0.114) | 0.569 |  | –0.153 (0.104) | 0.145 |  | –0.155 (0.058) | 7.62E–03 |  | –0.078 (0.086) | 0.366 |
| cg05825244 | 20 | 0.125 (0.036) | 6.51E–04 |  | 0.252 (0.117) | 0.034 |  | 0.103 (0.107) | 0.339 |  | 0.224 (0.061) | 2.90E–04 |  | 0.111 (0.098) | 0.261 |
| cg24263062 | 20 | 0.099 (0.036) | 6.18E–03 |  | 0.254 (0.107) | 0.020 |  | 0.232 (0.103) | 0.028 |  | 0.142 (0.059) | 0.017 |  | 0.053 (0.097) | 0.584 |
| cg01523712 | 20 | 0.076 (0.039) | 0.052 |  | 0.230 (0.121) | 0.063 |  | 0.045 (0.110) | 0.683 |  | 0.313 (0.068) | 7.96E–06 |  | 0.205 (0.105) | 0.054 |
| cg09349128 | 22 | –0.124 (0.031) | 8.33E–05 |  | 0.006 (0.062) | 0.922 |  | –0.062 (0.083) | 0.459 |  | –0.082 (0.042) | 0.053 |  | –0.160 (0.081) | 0.050 |
| cg15376401 | X | 0.090 (0.037) | 0.016 |  | 0.177 (0.081) | 0.034 |  | 0.003 (0.080) | 0.971 |  | 0.149 (0.048) | 2.35E–03 |  | 0.231 (0.096) | 0.018 |
| Note: DFTJ, participants selected from Dongfeng-Tongji cohort; ACS-WH, ACS patients recruited from Wuhan; ACS-GD, ACS patients recruited from Zhuhai; WHZH, residents selected from the Wuhan-Zhuhai Cohort; SY, individuals recruited from Shiyan, China. Abbreviations: Chr, Chromosome; SVs, surrogate variables. | | | | | | | | | | | | | | | |
| *^a^*Estimates were calculated by linear regression models with inverse-normal transformed methylation values as the dependent variable, inverse-normal transformed plasma copper values as the independent variable. DFTJ: adjusted for age, sex, smoking status, drinking status, BMI, neutrophils, the proportions of lymphocytes, monocytes, eosinophils, basophils cell and SVs; ACS-WH: adjusted for age, sex, smoking status, drinking status, BMI, the proportions of neutrophils, lymphocytes, intermediate cell and SVs; ACS-GD: adjusted for age, sex, smoking status, drinking status, BMI, the proportions of neutrophils, lymphocytes, intermediate cell and SVs; WHZH: adjusted for age, sex, smoking status, drinking status, BMI, region indicator, the proportions of neutrophils, lymphocytes, intermediate cell and SVs; SY: adjusted for age, sex, smoking status, drinking status, BMI, the proportions of neutrophils, lymphocytes, monocytes, eosinophils, basophils cell and SVs. | | | | | | | | | | | | | | | |

| Table S3. Significantly enriched *KEGG* pathways (*FDR* <0.05) associated with genes annotated to the top 500 CpG sites. | | | | |
| --- | --- | --- | --- | --- |
| KEGG Pathways | Genes harboring the 500 CpGs | | | |
|  | n | Ratio (%) | *p*-Value | *FDR* |
| Insulin signaling pathway | 8 | 5.84 | 5.21 ×10^–5^ | 7.66×10^–3^ |
| Type II diabetes mellitus | 5 | 10.64 | 8.23×10^–5^ | 7.66×10^–3^ |
| Amyotrophic lateral sclerosis | 5 | 9.43 | 1.47×10^–4^ | 9.11×10^–3^ |
| Calcium signaling pathway | 8 | 4.52 | 3.06×10^–4^ | 1.42×10^–2^ |
| Purine metabolism | 7 | 4.40 | 8.45×10^–4^ | 3.14×10^–2^ |
| Note: n represents the number of genes within our top 500 sites. Ratio= (the number of genes within our top 500 sites/the total genes in the *KEGG* path) ×100%. | | | | |
| Abbreviations: CpG, cytosine-phosphate-guanine methylation site; *FDR*, false discovery rate; *KEGG*, Kyoto Encyclopedia of Genes and Genomes. | | | | |

| Table S4. Meta-analysis of associations between four plasma copper-related CpGs (*FDR* <0.05) with major cardiovascular risk factors. | | | | | | | | | | | | | | | | |
| --- | --- | --- | --- | --- | --- | --- | --- | --- | --- | --- | --- | --- | --- | --- | --- | --- |
| CpG | BMI | | SBP | | DBP | | HDL-C | | LDL-C | | TG | | Glucose | | CRP | |
|  | metaZ | *p*-Value | metaZ | *p*-Value | metaZ | *p*-Value | metaZ | *p*-Value | metaZ | *p*-Value | metaZ | *p*-Value | metaZ | *p*-Value | metaZ | *p*-Value |
| cg05825244 | 1.91 | 0.06 | 0.33 | 0.74 | 0.30 | 0.76 | –2.79 | 0.005 | –1.61 | 0.11 | –0.83 | 0.40 | 0.71 | 0.48 | 2.13 | 0.03 |
| cg20995564 | –1.66 | 0.10 | 1.21 | 0.23 | 1.16 | 0.25 | 1.42 | 0.15 | –1.75 | 0.08 | 0.84 | 0.40 | 0.70 | 0.49 | –1.62 | 0.11 |
| cg18608055 | –1.73 | 0.08 | –0.52 | 0.60 | –0.96 | 0.34 | 0.37 | 0.71 | 0.48 | 0.63 | –0.02 | 0.98 | 0.79 | 0.43 | 1.01 | 0.31 |
| cg26470501 | –0.98 | 0.32 | 0.50 | 0.62 | 0.25 | 0.80 | 0.78 | 0.44 | –1.47 | 0.14 | –0.62 | 0.54 | –1.15 | 0.25 | –2.98 | 0.003 |
| Note: Only nominally significant associations were presented. All methylation-traits association was analyzed separately in Wuhan healthy residents from the WHZH panel, Zhuhai healthy residents from the WHZH panel and the SY panel, with adjustment of age, sex, smoking, drinking and BMI, except for itself, and the DFTJ panel with adjustments of age, sex, smoking, drinking, BMI and ACS indictor, except for itself, and then combined using a meta-analysis. | | | | | | | | | | | | | | | | |
| Abbreviations: ACS, acute coronary syndrome; BMI, body mass index; Chr, Chromosome; CRP, C-reactive protein; DBP, diastolic blood pressure; HDL-C, high-density lipoprotein cholesterol; LDL-C, low-density lipoprotein cholesterol; SBP, systolic blood pressure; TG, triglycerides. | | | | | | | | | | | | | | | | |
