# Supplementary material for: Profile of copper-associated DNA methylation and its association with incident acute coronary syndrome
Source: Clin Epigenetics. 2021 Jan 27;13:19. doi: 10.1186/s13148-021-01004-w (PMC7839231; doi:10.1186/s13148-021-01004-w)
Supplement: Supplementary file 3 — Additional file 3. Figure S1: The flowchart of the study. Figure S2: Distribution of plasma copper concentrations in five panels. Figure S3: Regional association plot of Cu meta-analysis results within and surrounding the EBF4 gene. [file 13148_2021_1004_MOESM3_ESM.docx]

**Additional file 3.**

**Profile of copper-associated DNA methylation and its association with incident acute coronary syndrome**

Pinpin Long^†^, Qiuhong Wang^†^, Yizhi Zhang, Xiaoyan Zhu, Kuai Yu, Haijing Jiang, Xuezhen Liu, Min Zhou, Yu Yuan, Kang Liu, Jing Jiang, Xiaomin Zhang, Meian He, Huan Guo, Weihong Chen, Jing Yuan, Longxian Cheng, Liming Liang, and Tangchun Wu^*^

**Figure S1.** The flowchart of the study.

**Figure S2.** Distribution of plasma copper concentrations in five panels.

**Figure S3.** Regional association plot of Cu meta-analysis results within and surrounding the *EBF4* gene.


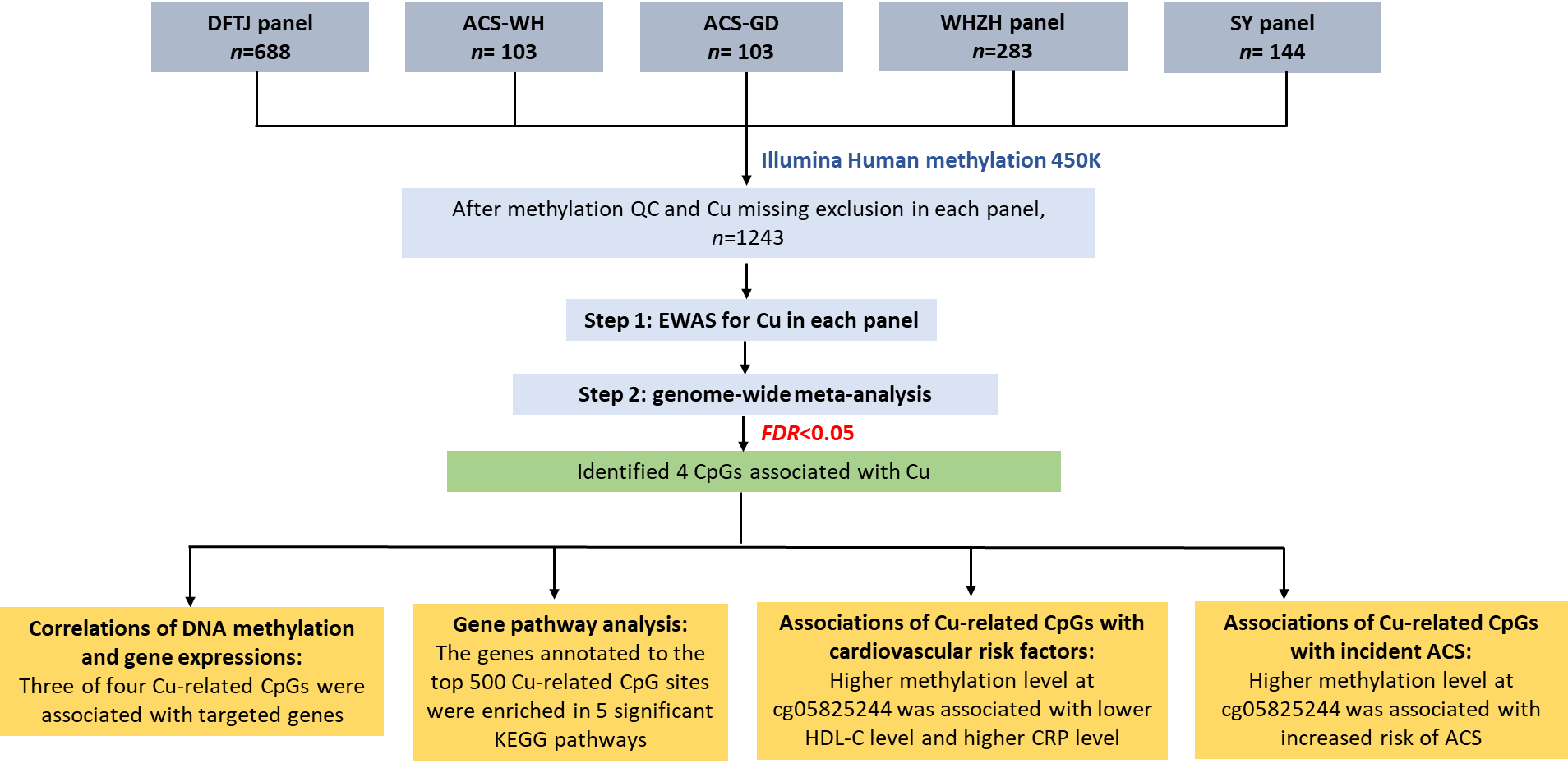


**Figure S1.** The flowchart of the study. ACS, acute coronary syndrome; ACS-WH, ACS patients recruited from Wuhan; ACS-GD, ACS patients recruited from Zhuhai; CpG, cytosine-phosphoguanine sites; Cu, copper; CRP, C-reactive protein; DFTJ, participants selected from Dongfeng-Tongji cohort; EWAS, epigenome-wide methylation study; HDL-C, high-density lipoprotein cholesterol; SY, individuals recruited from Shiyan, China; WHZH, residents selected from the Wuhan-Zhuhai Cohort.

**
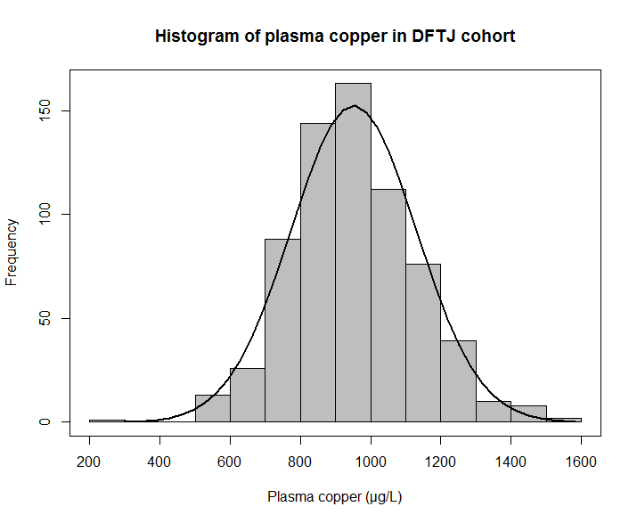

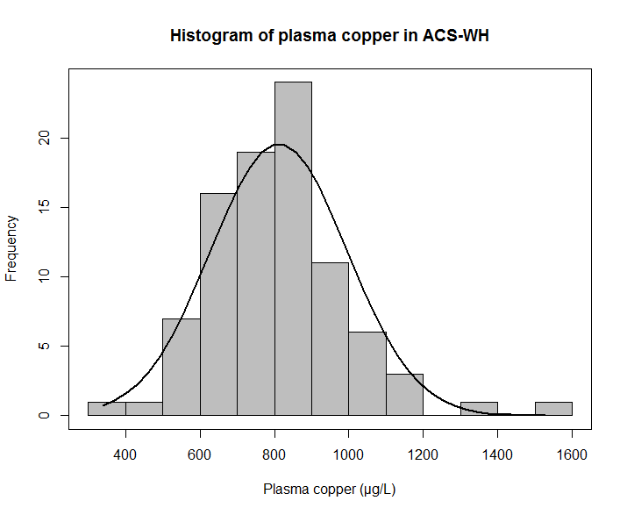

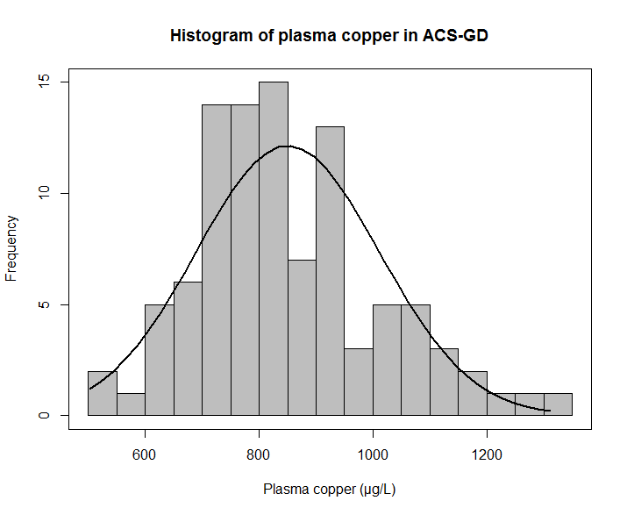
**


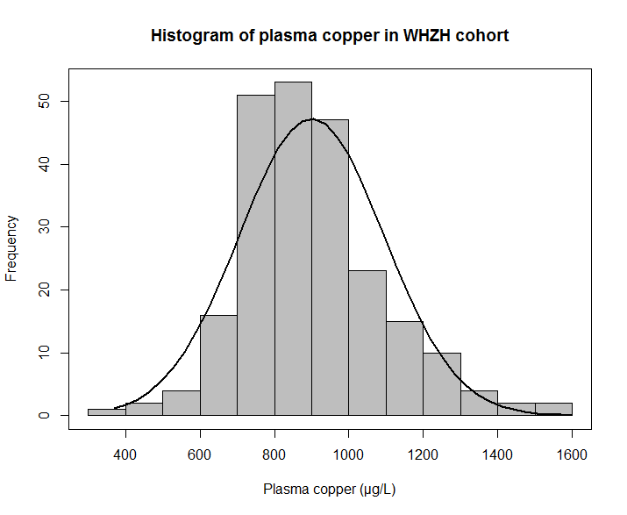

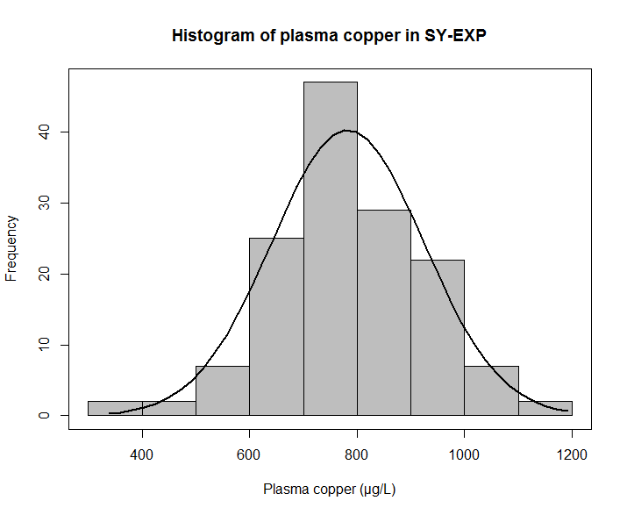


**Figure S2.** Distribution of plasma copper concentrations in five panels.


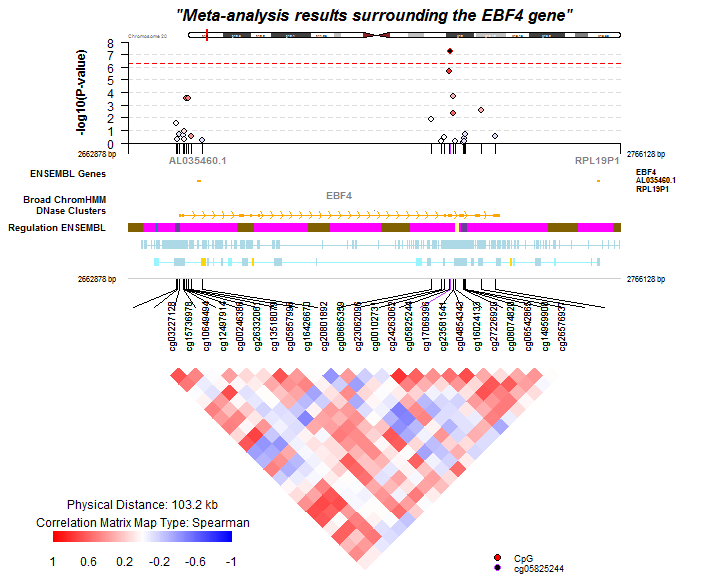


**Figure S3.** Regional association plot of Cu meta-analysis results within and surrounding the *EBF4* gene. The x-axis depicts the position in base pair (bp) (hg38) for the entire *EBF4* gene region. The y-axis indicates the strength of association in terms of negative logarithm of the association *p*-Value. Each circle represents a CpG site. Red dashed line within the graph indicates the genome-wide significance threshold. The regulatory information and correlation matrix of other CpG sites in the region with the top hit are shown below the x-axis. Color intensity marks the strength of the correlation and color indicates the direction of the correlation.
